# Preclinical models of atherosclerosis: An overview

**DOI:** 10.22038/IJBMS.2024.74352.16148

**Published:** 2024

**Authors:** Priyanka Arya, Vikram Sharma, Surabhi Thapliyal, Rahul Sagar, Priyanka Singh

**Affiliations:** 1 Galgotias College of Pharmacy, Greater Noida, U.P., India; 2 Department of Pharmacology, All India Institute of Medical Sciences, Rishikesh 249203, India; 3 AnovIP, New Delhi, India

**Keywords:** Atherosclerosis, Cardiovascular disease, Diets, Inflammation, Low-density lipoprotein

## Abstract

Atherosclerosis is a primary cause of illness and death globally and its mechanism is still unclear. Different animal models have been created to evaluate the progression of atherosclerosis, allowing researchers to carefully control the circumstances of the experiment as well as the nutrition and environmental risk factors. To investigate the negative effects of various interventions, pathophysiological alterations might be generated utilizing genetic or pharmacological methods. These models’ molecular and pathophysiological mechanisms have been clarified through experiments, and they have served as platforms for the creation of new drugs. Different models can be employed to address various research problems, each with its own benefits and drawbacks. In the current review study, various species of atherosclerosis models are discussed, along with the viability of using them in experiments.

## Introduction

Cardiovascular diseases (CVDs) are still some of the main causes of premature deaths and are the cause of increased medical expenses (1). CVDs consist of atherosclerosis, ischemic heart disease, stroke, heart failure, peripheral arterial disease, and several other cardiac and vascular disorders, which are major contributors to the decline in quality of life and death worldwide (2). 

Atherosclerosis is a chronic disease in which arteries harden through the accumulation of plaque in the subendothelial space of arteries. The phenomenon is characterized by vascular wall lipid accumulation, scarring, and inflammation associated with the intimal space of arteries which leads to thickening of the vascular wall, calcification, and in some cases thrombosis (3). This procedure underlies various clinical manifestations including - coronary artery disease (CAD), myocardial infarction (MI), peripheral vascular disease (PVD), abdominal aortic aneurysms (AAA), ischemic gangrene, and several cases of heart failure and stroke (4). 

When cholesterol-containing low-density lipoproteins build up in the intima, the artery’s innermost layer, and endothelial function is disrupted, the atherosclerotic process begins. As a result, leukocytes, adhesion molecules, and chemokines encourage the recruitment of monocytes and T cells as well as the transmigration of inflammatory cells. Monocyte and T-cell recruitment is facilitated by leukocyte adhesion molecules and chemokines (5). Scavenger receptors and toll-like receptors, two types of pattern recognition receptors, are activated by monocyte differentiation into macrophages. Lipoprotein internalization is mediated by scavenger receptors, and this results in the development of foam cells (6). Toll-like receptors transmit activating signals that lead to the generation of various cytokines, chemokines, growth factors, proteases, and vasoactive molecules which contribute to local inflammation and growth of the plaque. Intensified inflammatory activation may lead to local proteolysis, impairment of cholesterol transport, plaque rupture, and thrombus formation, which results in heart attack, stroke, and peripheral vascular disease (7).

An ideal animal model of atherosclerosis is one that closely resembles human anatomy and pathophysiology and can be used in medical and pharmaceutical research to produce results that can be applied to human medicine (8). In addition, it must be readily available, cheap to maintain, controllable, and able to share the topography of the damage with humans. Animal models of atherosclerosis are often associated with increased plaque formation due to a high-cholesterol Western diet, genetic modification of genes involved in cholesterol metabolism, and increased risk factors for atherosclerosis. In animal models of atherosclerosis, lesions form either spontaneously or in response to interventions such as diet, mechanical, pharmacological, or immunological stimulation (9).

Mice and rabbits were the most often utilized animal models, followed by pigs and non-human primates. Each of these models has an individual set of benefits and drawbacks. The mouse has evolved into the preferred species for research in the development of atherosclerosis experimentally due to its quick reproductive rate, genetic manipulation, and capacity to monitor atherogenesis in an adequate frame of time (10)(Table 1).

The C57BL/6 strain is more prone to atherosclerosis, with hypercholesterolemia (about 200 mg/dl) and scattered fatty stripe lesions after feeding an atherogenic diet and thus the most extensively used (11). Preclinical models can be used to investigate the basic mechanisms behind the atherosclerotic disease process, as well as the impact of dietary or other interventions on disease progression or reversal, all while maintaining a controlled environment (12). The effect of risk factors on the development of atherosclerotic disease has been studied in animal models, which has the advantage of removing other factors. Throughout these operations, considerable examination of processes occurring in the artery wall has yielded useful information regarding diagnostic and treatments using animal models (12-13). The present review highlights the different species of atherosclerosis models, along with the potential benefits of using them in experiments that provide insight into the mechanisms that contribute to illness progression and regression in humans.

***Methodology***

Online search engines and databases such as Science Direct, Scopus, PubMed, and Google Scholar were used to obtain scientific literature. Keywords were used to search “Cardiovascular disease”, “Atherosclerosis”, “Zymosan”, “Diet models “Genetically modified mice”, “Inflammation” “Aortic Plaque”, “Lipid profile”, “Rabbit models”

***Animal models of atherosclerosis***

**Diet-induced atherosclerosis**

**Figure 1:**
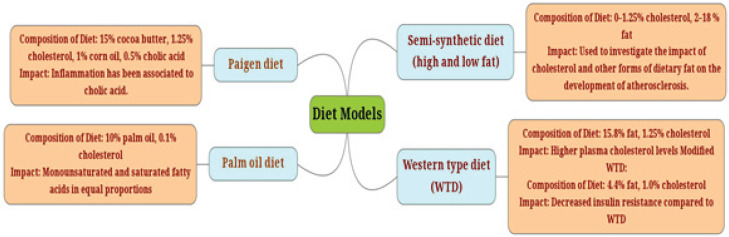
Murine atherogenic diet models are shown along with their compositions and their impact on atherosclerosis development

One of the most important environmental factors linked to the occurrence of cardiovascular illnesses is dietary fat. The development of atherosclerosis has been linked to the high-cholesterol and saturated-fat diets. The development of experimental atherosclerosis in mice appears to need the production of persistent hypercholesterolemia to levels of 300 mg/dl (14). A variety of diets have been used, varying in cholesterol levels, fatty acid levels and types, and the presence or absence of cholate (Figure 1). The effects of these constituents on the expression of hepatic genes involved in lipid homeostasis have been detected in some cases (15).

Diet, inflammation, and lipid metabolism have all been linked to the development of atherosclerosis. The mechanisms involved in the Western food pattern increase circulating inflammatory indicators and raise the risk of coronary heart disease are not known (16). Evidence from diet-induced atherosclerosis in mice reveals that innate immune receptors drive inflammation. TLR2 and TLR4 are two toll-like receptors that are likely to be implicated. Human feeding studies revealed that eating high-fat diets activates TLRs and up-regulates various markers of cardiovascular risk, comprising cholesterol, low-density lipoprotein (LDL), and inflammatory markers (17)(Figure 1).

***HFD Composition***

Composition of standardized high-fat diet (g/kg diet): Casein 244.4; L-Cystine 3.67; Soybean oil 61.1; Lard 177.4; Corn starch 112.28; Maltodextrin 161.3; AIN-93 vitamin mix 12.2; Sucrose 122; Choline chloride 1.7; AIN- 93G mineral mix 42.8; Cellulose 61.1; and t-BHQ 0.05 (15-16).

***Role of ingredients of HFD***

1. Casein: Casein has the ability to increase the body’s fat absorption (18).

2. Cystine: Cystine promotes fat storage in the liver (19). 3. Lard: Lard has about 39% saturated fatty acids, 45% monounsaturated fatty acids, and 11% polyunsaturated fatty acids (20).

4. Corn starch, maltodextrin, and sucrose: Corn starch, maltodextrin, and sucrose are carbohydrates and they provide energy. As excess glucose travels through the blood vessels, it weakens the arterial walls, making them leaky and dysfunctional, which can lead to atherosclerosis (21).

5. AIN-93 G mineral mix and AIN-93 vitamin mix: The minerals as well as vitamins complete the energy requirements of the body by acting as co-enzymes, participating in the metabolic processes. Vitamins, e.g., thiamine and niacin are involved in protein and carbohydrate metabolism as well as providing energy from carbohydrates. Intake of vitamins plays an important role in avoiding atherosclerosis (e.g., vitamins B2, B3, B5, B6, vitamin C, and Vitamin D)(22). In atherosclerotic conditions, the level of vitamins and minerals is found low as reported in research findings. Minerals, e.g., manganese are also involved in the metabolism of carbohydrates, fats, and proteins and play a crucial role in insulin activity and metabolic disorders (23).

6. Choline chloride: choline chloride is an essential component for fat metabolism (24).

7. t-BHQ: it acts as a preservative in animal diet and also has antioxidant properties (24).

**Zymosan-induced atherosclerosis model**

Zymosan A (Zym) is a glucan comprising 1, 3-glycosidic linkages connecting repeated glucose units. It stimulates the immune system via activation of TLR 2 and dectin 1 (25). Zym is a ligand that can be found on the surface of fungi such as yeast. Zym is a boiling, trypsin-treated *Saccharomyces cerevisiae* cell wall preparation that has been utilized for over 50 years as a model microbial particle in studies on innate immune responses such as inflammatory cytokine release, phagocytosis processes, and complement activation (26). It is in the powder form with minute bulges and appears off-white to light brown in shade and its storage temperature is 2-8 ^°^C (27).

Inflammation plays a crucial role in the development of animal models for disorders like acute peritonitis, rheumatoid arthritis, atherosclerosis, insulin resistance, and multiple organ failure (27). Accumulated evidence suggests that the immune system is stimulated by activation of TLR2 and TLR6 with Zym and a high-calorie diet (28). Activation of Zym-induced TLR2 signaling pathways is associated with vascular inflammation in atherosclerosis, which further transmits transmembrane signals that lead to activation of NF-ƙB (29). NF-ƙB is a serious transcription factor for the induction of inflammatory cytokines and also changes lipid metabolism by interference of LDLR, which impairs cholesterol transport and forms foam cells, which leads to plaque buildup in the arterial wall. This, in turn, increases the risk of atherosclerosis by disturbing the blood flow of vascular arteries (30).

Zhang and coworkers stated that administration of Zym in rats impairs blood lipid levels and releases the inflammatory mediators that induce atherosclerosis development (31). Feingold *et al*. stated that giving Zym (80 mg/kg) to C57BL/6 mice together with a high-cholesterol diet induces inflammation which impairs the lipid and lipoprotein metabolism by increasing PCSK9 levels and decreases hepatic LDLR levels (32). Liu and coworkers demonstrated that the administration of Zym in rabbits along with a raised cholesterol diet induces atherosclerosis by activation of the inflammatory signaling path (33). Furthermore, research reported that administration of Zym in Wistar rats induces atherosclerosis via activation of focal adhesion kinase (FAK) and the Nf-ƙB pathway which in turn form foam cell formation and results in lipid accumulation in the aorta leading to atherosclerosis (34). Arya *et al.* in 2020 reported that zym along with a high-fat diet is a good preclinical model for the development of atherosclerosis. They provide evidence that intraperitoneal injection of 80 mg/kg in Wistar rats shows increased TLR2, Nf-ƙB, and TNF-α, decreased levels of LDLR, altered serum levels of cholesterol, and histopathological increase in aortic plaque level (16). This is a recent preclinical model for atherosclerosis development which will contribute to understanding the progression of atherosclerosis and its clinical consequences, and that allows significant improvement in treatment (35).

**Genetically modified atherosclerosis mice models**

*Apolipoprotein E deficient (ApoE-/-) mice*

**Figure 2:**
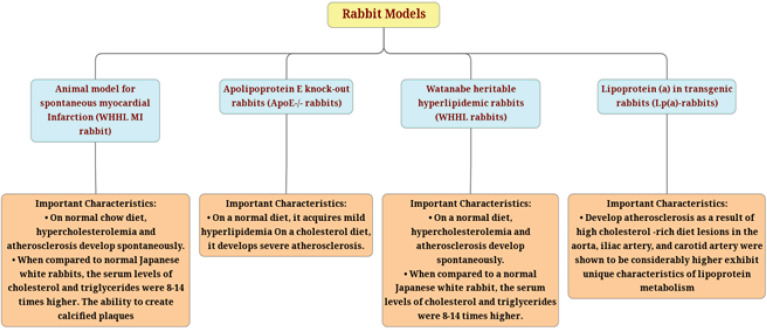
Outline of rabbit models. The figure depicts the important features, benefits, and drawbacks of each model

 The first ApoE-/- mice were created almost simultaneously in two laboratories in 1992. To delete the ApoE gene in mouse embryonic stem cells researchers used the homologous recombination technique (36). The advantages of ApoE-/- mice are that they are healthy, weigh similar to wild-type mice, and reproduce at the expected rate. In the lipid profile researchers revealed a crucial modification in their deviant counterparts. When ApoE-/- mice were fed a normal diet it was observed that their ability to maintain the plasma lipoproteins was severely decreased, subsequent to plasma cholesterol levels of 400-600 mg/dl, however, wild-type mice have 75-110 mg/dl levels (37). Increase in VLDL-sized particles is responsible for this drastic change. The development of significant hypercholesterolemia suggests that deficiency of ApoE is sufficient to cause massive changes in lipoprotein metabolism in the absence of an environmental stimulus when fed a normal diet. In another study, sensitivity to dietary fat and cholesterol in the absence of ApoE increased (Figure 2). In mice of the wild type, it was observed that the plasma cholesterol levels were doubled after several weeks of feeding a Western-type diet consisting of 21% fat and 0.15 percent cholesterol, which is similar to the everyday diet of Western countries, while in mice lacking the ApoE gene, an increase in total plasma cholesterol is seen (38).

Researchers reported that mice lacking apolipoprotein E spontaneously had higher levels of total plasma cholesterol and triglycerides and lower levels of high-density lipoprotein. The mice experience a time-dependent development of arterial lesions. When mice were young, the lesions were concentrated in the aortic sinus; however, by the time the mice were 8 to 9 months old, the lesions had spread broadly throughout the arterial tree. Subendothelial foam cell deposits were seen near the attachment points for the valve in the aortic sinus of young mice. Foam cell deposits, unbound cholesterol, and mixed smooth muscle cells made up the atherosclerotic lesions by the age of 5 months. The vascular lesions were more complex after 8 to 9 months of age, and fibrous cap lesions were also evident. Transmission electron microscopy showed foam cells, aortic plaque, and cell debris (39).

 The series of incidences that result in plaque development in ApoE-/- mice is strikingly similar to that observed in well-researched bigger animal models of atherosclerosis as well as in humans (36). Although this mouse model is used by many research teams, it has some drawbacks. For instance, the multifunctional protein apoE influences migration, oxidation, smooth muscle growth, and the transfer of reverse cholesterol by macrophages. The above functions may influence the development of atherosclerotic plaques in ApoE-/- mice, irrespective of plasma lipid levels. This model mimics the morphology of human plaque, which is a significant improvement from conventionally physically produced atherosclerosis (39). 

*LDL receptor-deficient (LDLr-/-) mice*

In 1993, mice with a specific LDL receptor inactivation were produced. When feeding a typical diet to LDLr-/- mice, investigators found slightly higher plasma cholesterol levels compared with wild-type mice and no or only moderate atherosclerosis development. IDL and LDL particle rise was more pronounced, but HDL and triglycerides were unaltered in terms of lipoprotein particles (40).

The cholesterol aggregates primarily in large lipoprotein particles such as chylomicron remnants, bad cholesterol LDL, and VLDL, when compared to ApoE-/- mice. A Western-type diet results in higher and more advanced lesions that have a collagen-rich fibrous cap, a necrotic core with cholesterol clefts, and improved cellular structure adjacent to the lumen (41). Time-dependent plaque formation occurs, starting in the proximal aorta and moving to the distal aorta. Much like in humans, atherosclerotic lesions are more prevalent in places where blood flow is impaired. Comparing the LDLr-/- mouse model to ApoE-/- mice, there are a few benefits. In the beginning, plasma cholesterol is primarily carried by LDL components, leading to a more human-like lipid profile. Second, unlike ApoE deficiency, inflammation is unaffected by the absence of LDL receptors (42)(Table 1). As a result, the development of atherosclerotic plaques in this mouse model is predicated on high plasma lipid levels rather than other functions associated with the LDL receptor. Finally, the LDLr-/- mouse model exhibits the same traits as human familial hypercholesterolemia, which is caused by the lack of functional LDL receptors (Figure 2)(42). 

*ApoE/LDL receptor double-knockout mice*

ApoE/LDL receptor double knock-out mice were introduced immediately after ApoE-/- and LDLr-/- mice, and they represent a model that develops more severe hyperlipidemia and atherosclerosis than the previous two (43). It is an animal model with spontaneous atherosclerotic plaque development, and it has been shown that in ApoE/LDL receptor double knock-out mice, the progression of atherosclerosis is usually more evident than in mice deficient for ApoE alone, even on a typical chow diet**. **Except for the considerable elevations in B48 and B100 apolipoproteins, there is no substantial difference in the lipoprotein profile of the double knockouts compared to ApoE-/- mice; they both exhibit high levels of VLDL and LDL (44). Without the use of an atherogenic diet, this mouse model is thought to be useful for studying the anti-atherosclerotic effects of potential treatments (Figure 2).

*ApoE3-Leiden mice*

Although ApoE-/- mice and LDLr-/- mice are the most widely utilized animal models for atherosclerosis, ApoE3-Leiden mice are also used in many research studies. Apolipoprotein (Apo) E3-Leiden is linked to a genetic form of hyperlipidemia and is found in a Dutch family. Transgenic mice were developed using a genomic 27-kilobase DNA construct taken from the APOE3-Leiden proband, which included the ApoE gene, ApoC1 gene, and all regulatory components. This was done to examine the effects of the ApoE3-Leiden mutation *in vivo* (45). Surprisingly, while being less prone to atherosclerosis than ApoE-deficient mice, these animals have much higher total plasma cholesterol and triglyceride levels when fed a Western-style diet. This is mostly due to an increase in VLDL/LDL particles, demonstrating that ApoE3-Leiden mice have a lipoprotein profile similar to that of humans. Another advantage is the ability of ApoE3-Leiden mice to manufacture functional ApoE (15). This allows researchers to investigate the impact of higher plasma lipid levels without disrupting inflammatory processes, which is a major drawback of the ‘traditional’ ApoE-/- mouse paradigm. ApoE3-Leiden mice are being employed as a model to investigate aspects involved in ApoE metabolism and, in particular, the etiology of familial dyslipidemia. Furthermore, ApoE3-Leiden mice are being used to investigate the problems of venous bypass grafting, a treatment that bypasses the artery blocked by atherosclerosis (Figure 2)(8).

*PCSK9-AAV mice*

A new line of mice models without germline genetic modification is emerging in the field of atherosclerosis research, in addition to the aforementioned models. In 2014, two research groups separately described the so-called pro-protein convertase subtilisin/kexin type 9 (PCSK9)-adeno-associated virus (AAV) mice as a quick, adaptable, and cost-effective animal model for atherosclerosis (46). PCSK9, a newly discovered human subtilase, is a serine protease that is abundantly expressed in the liver and has plasma concentrations of 100 to 200 ng/ml. PCSK9 has been found in several studies to inhibit hepatic LDL absorption by enhancing LDL receptor endosomal and lysosomal degradation. In summary, circulating PCSK9 attaches to LDL receptors on the cell surface following maturation and secretion, and is then co-internalized with the receptor. This disrupts the receptor’s usual recycling mechanism to the plasma membrane, allowing it to degrade in the lysosome (47). In several animal models and humans, recombinant AAV vectors sustain long-term transgenic expression. Mice stably expressed PCSK9DY mRNA in the liver after a single intravenous injection of human D374Y or murine D377Y gain-of-function mutant PCSK9. Infection with the AAV virus has no negative consequences on the animals, and no symptoms of liver damage or immune response have been identified after infection (48). Total blood cholesterol in PCSK9DY-AAV transgenes animals was doubled 30 days after injection compared to control mice. In PCSK9^DY^-AAV mice, a Western-style diet aggravated hyperlipidemia, resulting in plasma cholesterol levels as high as 1165 mg/dl, whereas chow diet-fed mice barely reached 316 mg/dl. The lipoprotein profile of PCSK9^DY^-AAV mice fed a Western-type diet revealed an equal distribution of VLDL and LDL particles. In a dose-dependent manner, PCSK9^DY^ transgenic mice develop atherosclerosis. When Roche-Molina *et al*. combined PCSK9^DY^ expression with ApoE deficiency, the lesions doubled in size with no significant alterations in lipoprotein profile when compared to single mutants on the same diet (Figure 2)(49).

*Apolipoprotein E-deficient Fibrillin-1 mutant (ApoE-/-Fbn1C1039G +/-) mice*

**Figure 3:**
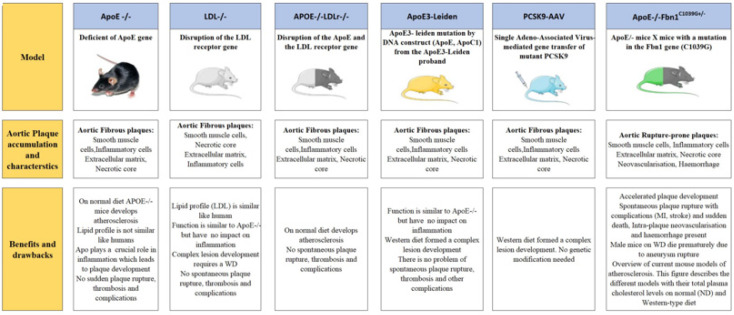
Outline of recent mouse models. The figure depicts the lipoprotein profiles, plaque features, benefits, and drawbacks of each model

Researchers reported that the cross-breeding of ApoE-/- mice with mice having a heterozygous mutation (C1039G+/-) in the fibrillin-1 (Fbn1) gene revealed the effect of altered elastin structure of the arterial wall on the advancement of atherosclerosis. Marfan syndrome is a hereditary condition characterized by the fragmentation of elastic fibers caused by mutations in the Fbn1 gene (50). As a result, increased arterial stiffening, raised pulse pressure, and gradual aortic dilatation are observed. Furthermore, the mutation causes very unstable plaques to form in ApoE-/- mice, leading to spontaneous plaque rupture and end-points such as myocardial ischemia and rapid death. Importantly, these occurrences do not or only infrequently occur in ApoE-/-Fbn1C1039G +/- mice fed a normal diet or in ApoE-/-Fbn1C1039G +/- mice provided a Western-type diet. This model highlights the role of elastin fragmentation as a requirement for atherosclerotic plaque rupture in mice when combined with a Western-style diet (Figure 3)(51).

**Rabbit models of atherosclerosis**

Atherosclerosis rabbit models have become less common since the year 2000, when Apolipoprotein E (ApoE) became available and LDL receptor knock-out mice have been developed (Table 1). The appeal of rabbit models can be explained by the fact that these animals are priced reasonably less, highly sensitive, and easy to maintain (Figure 2)(52).

**Pig atherosclerosis Model**

Pigs develop atherosclerosis, usually when fed a cholesterol-rich diet. The advantage of this model is that it has a similar lipoprotein profile to humans, developing atherosclerotic lesions in coronary arteries, and due to its large size, it’s easy to evaluate the non-invasive measurements of arteries as well as to take sufficient arterial tissue for the analysis of non-invasive measurements of arteries (53) (Table 1). Researchers have discovered three models for developing hypercholesterolemia and atherosclerosis in pigs without feeding an atherogenic diet with lipoprotein-associated mutations (Lpb5, Lpr1, and Lpu1). Moreover, it was also evident that not only the coronary artery but also the iliac and femoral arteries develop lesions that lead to the progression of disease (54).

*Non-human primates model*

Non-human primate species, when compared with other species, have the closest resemblance to humans. In the context of phylogeny, 98% of their genetic materials are similar (55). 

Rhesus and Cynomolgus monkeys: Rhesus monkeys developed complex atherosclerotic lesions in coronary arteries when fed a high-cholesterol and fat diet. The development of lesions was observed in the innermost layer of the blood vessel, i.e., the intimal layer, leading to thickening and increased density of vasa vasorum in the tunica media, which are characteristics also traced in humans. The regression of coronary atherosclerosis was first introduced via this model. Cynomolgus monkeys have been used on the grounds of their high sensitivity to a high-fat diet. When these monkeys were fed a high-cholesterol diet, it was revealed that their plasma and cholesterol levels were twice as high as those of rhesus monkeys, and they also increased lipid-loaded monocytes in the blood and foam cells, which led to plaque accumulation and faster disease progression (55).

The advantage of primates is that they are a great option because of their striking similarities to humans in terms of cardiac structure and function. Both species have abnormal cardiovascular physiology, as evidenced by lesion shape, plaque vulnerability, and the development of spontaneous luminal thrombosis (56)(Table 1). They have a comparable propensity for atherosclerosis, with younger animals showing a reasonable level of resistance to the disease’s development, but as they age, they are more likely to become vulnerable. Both species exhibit thrombosis (56). Monkeys have a comparable risk of developing atherosclerosis, with younger animals having a reasonable level of resistance development to atherosclerosis but being more likely to develop the disease as they age. Researchers also revealed that feeding high fat and cholesterol diet developed lesions similar to humans’ which was evidenced by similar histopathology features with humans (56).

Although they resemble humans significantly, primate models are less common than other various model types due to being more difficult to maintain because of their size, high cost, scarcity, and need for specialized housing facilities (55-56). Second, it takes a considerable amount of time to cause atherosclerosis. Beyond these, their broad usage is limited by the ethical issue surrounding the use of human-like monkeys in experiments. However, non-human primates are appropriate for accelerating the development of accurate biomarker devices for risk assessment and tracking the impact of medication therapies on the advancement of illness (57).

**Zebrafish model of atherosclerosis**

Zebrafish and human metabolism of lipids and lipoproteins are very comparable (58). All key nuclear receptors, lipid transporters, apolipoproteins, and enzymes involved in the metabolism of lipoproteins are expressed in zebrafish. There are various genetic mutant zebrafish models such as ldlr, lxr, and apo2. Zebrafish circulatory systems differ from those of mammals, yet they are comparable in development and structure (59). Researchers reveal that feeding a high-fat diet developed accumulation of lipid and cell infiltration in sections of the dorsal aorta (Table 1). The benefits of zebrafish, such as their tiny size, low cost of growing, high number of offspring, *ex utero* fertilization, and quick growth, make large-scale screening of potential targets both possible and economical. The drawback of this model is that only small amounts of blood can be taken from zebrafish older than 45 days of age (60).

**Table 1:** Comparing the models in terms of similarities and differences with the pathophysiology of atherosclerosis in humans

Model	Similarity	Differences	References
Mice	1. The C57BL/6J strain has been established as susceptible to atherosclerosis development and remains the most responsive one. 2. It has a predisposition toward T-helper 1-immunity and IFN (interferon)-γ production, which is a major atherogenic factor.	1. Lack of CETP, a protein that shuffles cholesterol between HDL and LDL in humans. 2. Intestinal absorption of cholesterol is also lower in mice compared with humans leading to higher endogenous cholesterol synthesis. 3. Humans are more likely to have lesions in the carotids, coronary arteries, and peripheral vessels like the iliac artery. Mice are more likely to have lesions in the aortic root, aortic arch, and innominate artery.	(10, 42, 43, 53)
Rabbits	1. Share similar lipid metabolism and lesion morphology observed in patients of homozygous familial hypercholesterolemia. 2. In addition, show more susceptibility of the male gender to coronary atherosclerosis.	1. Rabbit models predominantly show atherosclerotic lesions in the aortic arch and descending thoracic aorta, whereas coronary arteries and abdominal aorta are majorly impacted in humans. Moreover, plaque lesion is also found to be dissimilar to that of humans. 2. Lack apolipoprotein II and express CETP (cholesterol ester transfer protein). lack hepatic lipase in rabbits, resulting in triglyceride-carrying HDL (high-density lipoprotein) particles.	(52)
Pigs	1. Cardiac anatomy and physiology resemble closely those of humans. Atherosclerotic lesions exhibit advanced plaques and intraplaque hemorrhages.	1. Toxic diet is required to cause atherosclerosis in porcine.	( 53 - 54 )
Non-human primate models. Rhesus monkeys	1. Closest similarities to humans. The formation of complex atherosclerotic lesions in coronary arteries. which demonstrates intimal thickening and increased density of vasa vasorum in the tunica media, features also observed in humans. 2. Humanoid lipoprotein metabolism with a predominance of non-HDL lipoproteins, human-like HDL subclasses, and expression of CETP. 3. Upon feeding a high-fat diet, males develop more atherosclerosis than female animals, as is also the case in humans.	1. Limited for genetic modification	( 55 - 57 )
Zebrafish	1. Zebrafish and human metabolism of lipids and lipoproteins are similar.	1. Zebrafish circulatory systems differ from those of mammals, yet they are comparable in development and structure.	(58-60)

## Conclusion

The pathogenesis of cardiovascular diseases has been widely studied using animal models. There is no single, perfect animal model to represent every illness. The size, docility, simplicity of housing and reproduction, known genetic profile, affordability, and human-animal parallels are the main requirements for a suitable animal model **(**61**).** Smaller animal models, such as those of mice and rabbits, typically yield important insights into the pathophysiology and etiology of atherosclerosis. Knowing the natural course of atherosclerosis and its risk factors can help avoid illness. Larger animal models, on the other hand, are more accurate homologs of human illness, such as pigs and nonhuman primates. The established advanced lesions have histological characteristics that are comparable to those of people, ranging from an early fatty streak to an advanced lesion that involves ulceration and thrombus development. As a result, their application is more beneficial for the advancement of illness management, including the evaluation of the usefulness of imaging techniques and the effectiveness of pharmaceutical interventions (62).

## Authors’ Contributions

P A conceived the study and design and performed literature search. V S critically reviewed and approved the final manuscript. S T outlined the study and wrote the final manuscript. R S conceived the study and performed literature search and drafting. P S performed literature search and drafting. All authors approved the final manuscript.

## Future Perspectives

Growing evidence shows increased incidence and prevalence of cardiovascular diseases day by day and now it has become a challenging disease globally. The pathogenesis of one of these illnesses, atherosclerosis, has been studied throughout the past decade using a range of experimental techniques. As a result, atherosclerosis research has made considerable use of laboratory animals. Nowadays, transgenic animals are anticipated to clarify the cause-and-effect links in lesion development and progression, shedding light on the molecular etiology of atherosclerosis. All of the above-mentioned literature has significantly improved our understanding of this disorder.

## Conflicts of Interest

The authors declare that they have no conflicts of interest.
